# A DNA-Based Semantic Fusion Model for Remote Sensing Data

**DOI:** 10.1371/journal.pone.0077090

**Published:** 2013-10-08

**Authors:** Heng Sun, Jian Weng, Guangchuang Yu, Richard H. Massawe

**Affiliations:** 1 Department of Computer Science, College of Information Science and Technology, Jinan University, Guangzhou, People's Republic of China; 2 Key Laboratory of Functional Protein Research of Guangdong Higher Education Institutes, Institute of Life and Health Engineering, College of Life Science and Technology, Jinan University, Guangzhou, People's Republic of China; 3 International School, Jinan University, Guangzhou, People's Republic of China; NASA Jet Propulsion Laboratory, United States of America

## Abstract

Semantic technology plays a key role in various domains, from conversation understanding to algorithm analysis. As the most efficient semantic tool, ontology can represent, process and manage the widespread knowledge. Nowadays, many researchers use ontology to collect and organize data's semantic information in order to maximize research productivity. In this paper, we firstly describe our work on the development of a remote sensing data ontology, with a primary focus on semantic fusion-driven research for big data. Our ontology is made up of 1,264 concepts and 2,030 semantic relationships. However, the growth of big data is straining the capacities of current semantic fusion and reasoning practices. Considering the massive parallelism of DNA strands, we propose a novel DNA-based semantic fusion model. In this model, a parallel strategy is developed to encode the semantic information in DNA for a large volume of remote sensing data. The semantic information is read in a parallel and bit-wise manner and an individual bit is converted to a base. By doing so, a considerable amount of conversion time can be saved, i.e., the cluster-based multi-processes program can reduce the conversion time from 81,536 seconds to 4,937 seconds for 4.34 GB source data files. Moreover, the size of result file recording DNA sequences is 54.51 GB for parallel C program compared with 57.89 GB for sequential Perl. This shows that our parallel method can also reduce the DNA synthesis cost. In addition, data types are encoded in our model, which is a basis for building type system in our future DNA computer. Finally, we describe theoretically an algorithm for DNA-based semantic fusion. This algorithm enables the process of integration of the knowledge from disparate remote sensing data sources into a consistent, accurate, and complete representation. This process depends solely on ligation reaction and screening operations instead of the ontology.

## Introduction

As the hereditary basis of every living organism, DNA has an ability to store and process information. This information is determined by the sequence of four distinct bases (A, C, G, T). An oligonucleotide is a short, single-stranded DNA molecule, and the complementary base pairing enables hybridization into a double-stranded polymer. These features of DNA have inspired the idea of DNA computing [1]–[3]. DNA computing, known also under the name of molecular computing, has great advantages of in vivo computing and in vitro computing, such as massive parallelism, extraordinary information density and exceptional energy efficiency. In contrast to traditional silicon-based technology, DNA computing has the natural potential of semantic fusion and reasoning for big data.

Nowadays, ontology has gained more and more acceptance as one of semantic technologies to solve the problem of heterogeneous knowledge sharing [4]. Many research efforts have been devoted to ontology modeling over the past decade [5]–[9], and quite a few running systems based on manual ontologies have been developed [10]–[12]. However, data is accumulating at an astounding rate with increasing computing power. Many activities, for instance encoding an organism's DNA [13], collecting satellite data [14], and conducting scientific experiments at the Large Hadron Collider [15], can create a staggering amount of data. The growth of these big data outstrips the capacities of current ontology engineering practices and tools. In bioinformatics, the semantic integration of big data has been identified as a new frontier [16]. The same trend can also be observed in other scientific domains. For example, with a vast amount of geographical data becoming available from satellites, especially the recent opening of the Landsat archive [17], there comes an increasing demand for automatic semantic processing of remote sensing images (RSIs) in a reasonable amount of time. Up to now, reasoning from big data is challenging. As the winner of the Semantic Web Challenge, Williams provided the experimental results showing that reasoning over the Billion Triple Dataset required 3712 processors from IBM LS21 blade servers and the computation time was 1314 seconds per processor [18]. Although this dataset contains 898,966,813 triples and the size of the combined dataset is around 17 GB, the amount of data obtained from satellite devices and open sources on the Internet per day is much higher and beyond the capabilities of analyst to process the data with the help of ontology [19]. Novel tools and approaches are needed to address this problem that has arisen during the current period of rapid data and knowledge growth.

Now DNA computing has become an active research area [20]–[24]. DNA-based parallel computing takes advantage of many different DNA molecules to solve the NP-complete problems in polynomial or even linear time, while exponentially increasing time is required in silicon-based computer. In this paper, a DNA model is introduced for semantic fusion of the RSIs. It utilizes DNA computing and ontology technologies to enable the complete representation of the RSI's knowledge in linear time regardless of the amount of data obtained.

There is few published work in the literature about the application of DNA-based approach to semantic fusion. Tsuboi proposed a pattern matching algorithm based on stickiness of DNA molecules [25]. Semantic network technology is used to solve information recognition problem. However, the fusion of semantic relationship is not involved. This restricts the analysis and reasoning capacity of the processing system. Moreover, the encoding scheme in this algorithm is not suitable for arbitrary digital information and the different data objects have to be encoded by different oligonucleotides. However, an exhaustive representation is considered unrealistic. Church proposed a novel strategy to store digit information in DNA [20]. In Church's work, all data blocks can be programmed into a bitstream and then encoded onto thousands of oligonucleotides. But the sequential conversion code (Perl) faces the challenge from big data. Xu provided a new DNA computing model for graph vertex coloring problem [26], which can effectively reduce the solution space by seminested polymerase chain reaction. All these approaches described above lack support for semantic reasoning and little attention has been given to big data, which have become the key problems of knowledge sharing and semantic representation in the web environment.

In an attempt to overcome these difficulties, we propose here a novel DNA-based semantic fusion model as an extension of our previous research for distributed data application in remote sensing field [27]. In previous work, we have implemented a semantic fusion and reasoning system for the RSIs' retrieval. At present, the use of DNA computing in semantic fusion presents numerous opportunities for our future DNA reasoner. The inherent massive parallelism of DNA strands allows for big data storage and reasoning. The main efforts in this paper are to 1) develop a remote sensing data ontology with 1,264 concepts and 2,030 semantic relationships to annotate the RSIs; 2) encode arbitrary semantic properties, property values, semantic relationships and data types in DNA, and organize the semantic information into directed acyclic graph; 3) evaluate the performance of our parallel conversion method against the sequential approach with the Rest dataset [28]; 4) create an algorithm that takes advantage of the biochemical reaction to fuse the semantic information.

## Results and Discussion

### Remote sensing data ontology

Ontology, as a formal representation of both implicit and explicit domain knowledge, can help to deal with heterogeneous representations of data and their interrelationships. There exist several forms of ontology with different semantic richness. As a specification developed by World Wide Web Consortium, the Resource Description Framework (RDF) [29] can present semantic information of web resources. RDF Schema [30] provides a type system for RDF and defines classes and properties that may be used to describe classes, properties and other data resources. It can also be used to build a lightweight ontology by describing RDF vocabularies.

**Figure 1:**
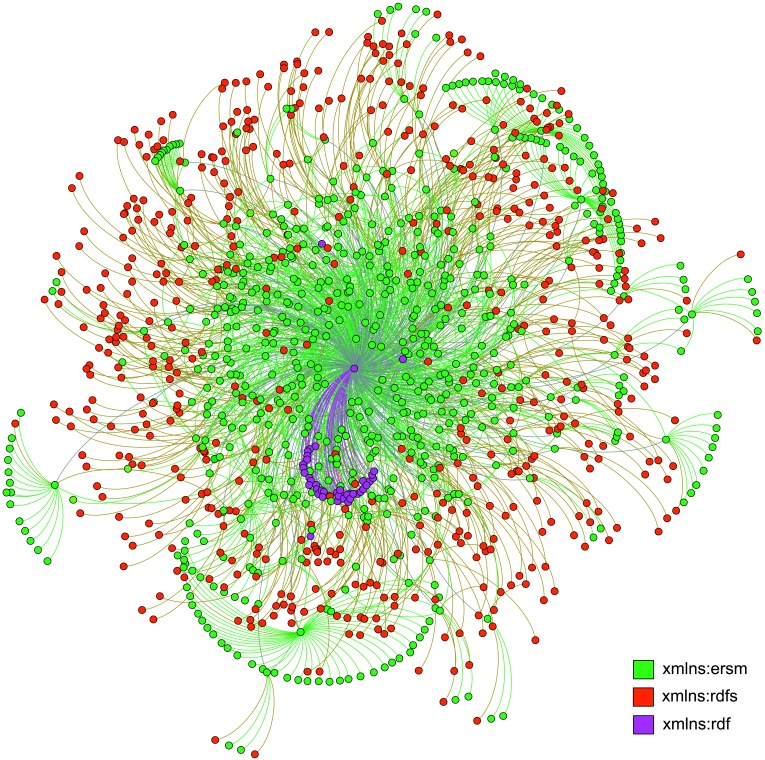
RDF graph of the remote sensing data ontology. This figure contains 1,264 nodes and 2,030 edges. Nodes are a set of classes and concepts in the remote sensing domain, such as *Worldwide_Reference_System*, *Multiple_Image_Alignment*, and *Spatial_Domain*, etc. Edges are a set of specific properties that characterize these classes. Classes, properties, and domains are all considered as ontology elements. All the elements are partitioned according to their namespaces. The namespaces in ontology vocabulary show the Uniform Resource Identifier References (URIrefs) as the URLs of web resources that provide further information about this vocabulary. The xmlns:ersm (http://cs.jnu.edu.cn/sun/ontology/ersm), xmlns:rdfs (http://www.w3.org/2000/01/rdf-schema), and xmlns:rdf (http://www.w3.org/1999/02/22-rdf-syntax-ns) are used mainly in our remote sensing data ontology. (For interpretation of the references to color in this figure, the reader is referred to the web version of this paper.)

Figure 1 illustrates the remote sensing data ontology by using RDF Schema language. The computer code of the ontology is provided in File S1. All terms in the ontology vocabulary are divided into five groups (namely, Identification Information, Data Quality Information, Spatial Data Organization Information, Instrument Information, and Location Information) to represent the content, quality, condition, and other characteristics of data. To enable the extensibility of the ontology, we evaluated the suitability of several existing geospatial metadata standards, including the Content Standard for Digital Geospatial Metadata: *Extension* for Remote Sensing Metadata [31], ISO 19115 [32] and ISO/TS 19319 [33]. The *Extension* defines the metadata elements published by the U.S. Federal Geographic Data Committee and documents digital remote sensing datasets in the US. While ISO 19115 does only provide a structure for describing digital geographic data and many elements in ISO 19115 are from the *Extension* standard. ISO/TS 19139 defines an XML schema implementation derived from ISO 19115. These two ISO standards are very simple but not suitable for ontology modeling. Considering the fact that the conceptual model in the *Extension* does not provide enough semantic description of geographic data, we construct a hierarchical structure of the ontology. The relationships among specific classes are encoded into the ontology structure. The RDF Schema properties *rdfs:range* and *rdfs:domain* describe the relationships between specific properties and classes, and a lot of image data relationships have been described using the domain properties from the *Extension* standard.

**Figure 2:**
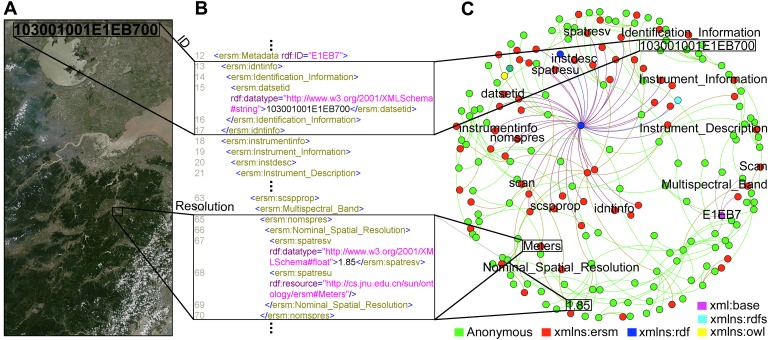
RDF instance description and visualization of an RSI. This figure includes three interactive parts: an RSI in A, an RDF annotation of the RSI in B, and data instance visualization in C. (A) One example RSI's ID is 103001001E1EB700 and its resolution is 1.85 meter. (B) The RDF identifies the data instance using the URIref and the image data can be described by making statements. A statement, such as “An RSI *103001001E1EB700* has a *nomspres* (Nominal Spatial Resolution) whose value is *1.85 meter*”, is represented by these two RDF/XML statement blocks. File S2 provides the complete RDF code of catalog ID *103001001E1EB700* imagery. (C) The 193 classes and concepts are partitioned into six colors according to their namespaces. Most of them (120 green nodes) represent blank nodes. They provide a way to more accurately make statements about data because constant values and most aggregate concepts may not have URIs. The other namespaces include xml:base (http://cs.jnu.edu.cn/sun/ontology/103001001E1EB700), xmlns:rdfs (http://www.w3.org/2000/01/rdf-schema), xmlns:ersm (http://cs.jnu.edu.cn/sun/ontology/ersm), xmlns:rdf (http://www.w3.org/1999/02/22-rdf-syntax-ns), and xmlns:owl (http://www.w3.org/2002/07/owl). (For interpretation of the references to color in this figure, the reader is referred to the web version of this paper.)

The real RSIs must be first preprocessed with semantic annotation technique, where semantic tags defined in the ontology are assigned to the phrases in the descriptive metadata of the RSIs. This facilitates the fusion and reasoning based on image semantics. RDF instance of an RSI is shown in Figure 2, where the metadata of RSI *103001001E1EB700* are annotated with the properties such as *imagequal* (image quality), *Cloud_Cover* and *spatresv* (spatial resolution value), etc. The property values are numerous “intermediate” anonymous resources to represent constant values (called literals) such as *Excellent*, *0*, *1.85*, or aggregate concepts such as RSI's structured *Nominal_Spatial_Resolution* values. Anonymous resources cannot be referred to from outside their defining RDF instance, and hence do not require meaningful names.

### Semantic property and data type

**Figure 3:**
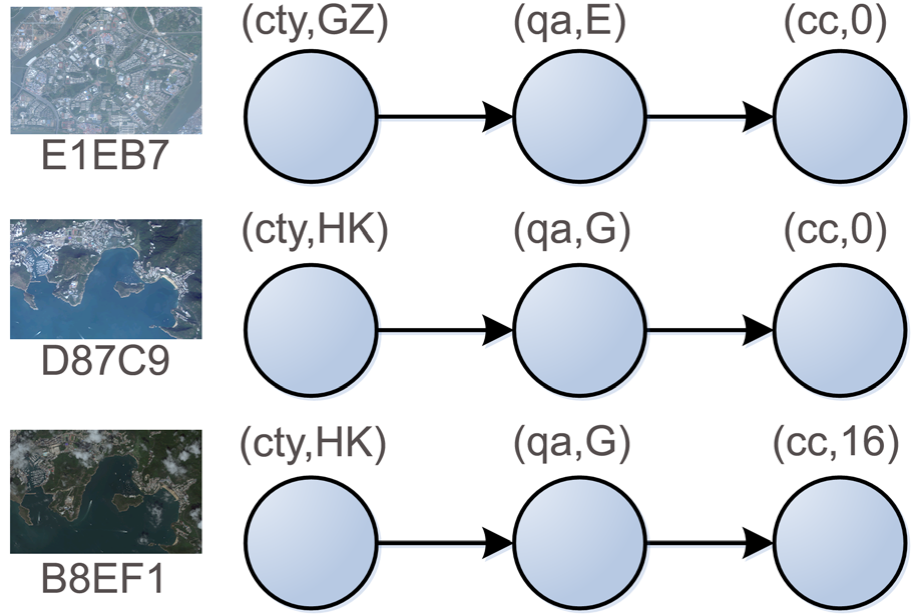
The linear model of semantic properties in three RSIs.

**Figure 4:**
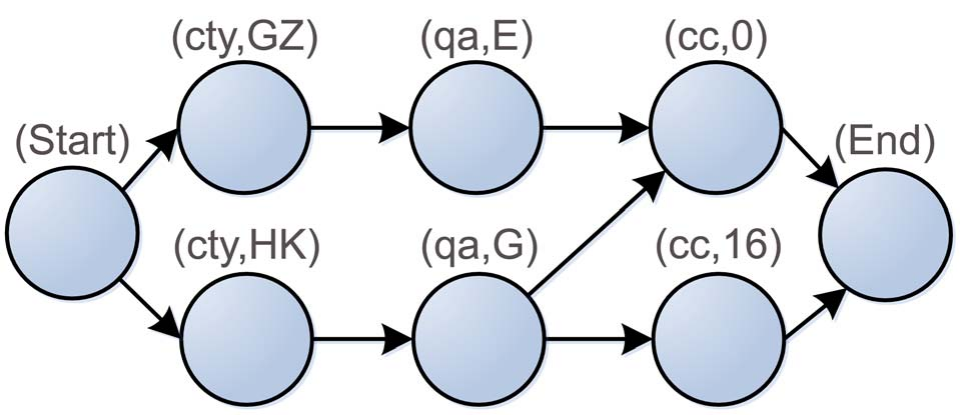
Network diagram of semantic property set.

In order to convert the classes and properties representing data semantics into the sequence of nucleotides, we propose the property representation and type design suited for DNA implementation. For example, this paper annotates three RSIs *E1EB7*, *D87C9* and *B8EF1* with three properties: *city* (*ct*), *imagequal* (*qa*) and *Cloud_Cover* (*cc*). The first image's property values are *Guang Zhou* (*GZ*), *Excellent* (*E*), and *0*, respectively. The other two's values are *Hong Kong* (*HK*), *Good* (*G*), *0*, and *HK*, *G*, *16*. Considering the linear structure of DNA strands, we arrange these properties and their values in sequence as shown in Figure 3. The label of a vertex is denoted as two-tuples (property name, property value). The edge denotes the connection between the vertices in the directed graph. To simplify the graphic structure, two new vertices labeled as “*Start*” and “*End*” are added to the directed graph and the vertices are integrated into one if they have the same property and property values. As shown in Figure 4, there are directed paths representing the annotation results of the RSIs between initial and terminal vertex in property network.

Everything would be simple if the type of property to be recorded was obviously in the form of the simple character string literal (plain literal) illustrated so far. However, most RSIs data involve structures that are more complex than that. Many constant values that serve as property values in the RSIs are numbers (e.g. the value of a *Nominal_Spatial_Resolution* property) or some other kinds of more specialized values. For example, Figure 4 illustrates a network diagram recording information about three RSIs, where the values of RSIs' *Cloud_Cover* property are literals “0%” and “16%”. However, there is no explicit indication that “0%” or “16%” should be interpreted as a number. The common practice in computer programming or database systems is to provide additional information about how to interpret a literal by associating a data type, such as integer, boolean, or string, with this literal. In our new DNA model, 4-nt oligonucleotides are used to provide this kind of information. Since DNA strand has no built-in data type system of its own, our model simply provides a way to explicitly indicate, for a given data type, what oligonucleotide should be associated with it. Table 1 shows the common data types. The data types in this model refer to the XML Schema Datatypes defined in [34]. An advantage of this approach is that it gives our model the flexibility to directly represent information obtained from various RSIs or web sources. It is worth noting that type conversions may still be required when moving data between systems having different sets of data types.

**Table 1:** Mapping from the data types to the oligonucleotides.

Data types	Oligonucleotides
string	TCGA
boolean	CTGA
float	GTCA
dateTime	AGTC
duration	TAGC
URI	ACGT
RName	GCTA
integer	CATG
undefined	TGCA

Moreover, a property value may sometimes appear to be simple, but may actually be more complex. For example, the unit information of the spatial resolution for satellite imagery is meter, but in some cases such information is not explicitly given and omitted in contexts where it can be assumed that anyone accessing the property value will understand the unit information being used. However, this assumption is generally unsafe in the wider context of the imagery. One might give a resolution value in kilometer or degree, whilst others might assume that is in meter. In general, a comprehensive consideration should be given to the explicit representation of unit information.

### Encoding the semantic information

Before the semantic information is converted into DNA, an encoding model is required. Although diverse coding strategies for DNA sequences have been developed and some have been demonstrated [20], [35], [36], no standard model exists. Church GM [20] first proposed a simple, universal strategy. In Church's work, arbitrary digital information can be converted into bitstreams by utilizing the ASCII code. These bits are then encoded onto the oligonucleotide library. Unlike conventional approaches, Church encodes one bit per base in order to meet the appropriate GC-content and introduces a 19-nt oligonucleotide to represent the data's address space.

However, the common type system is not considered in Church's encoding method. Thus, we propose a novel data encoding approach for semantic information. Firstly, the vertices and edges in Figure 4 are converted into DNA sequences in order to efficiently represent the semantic properties. Every vertex is associated with a 48-nt oligonucleotide which is denoted *V*. The full description about the mapping from the vertex property to the DNA sequence is provided in the Materials and Methods section. Now each *V*, except the *start* and *end* vertices, is decomposed into four oligonucleotides whose lengths are 24, 4, 4, 16: *V*  =  *NTUA*. *N*, *T*, *U*, and *A* represent the property name, data type, unit (or comment), and property value respectively. The unit value *U* depends on *N* and *T*. For example, the property name *cc* and property value *0* in the vertex (cc,0) are represented by the first and last parts of *V_(cc,0)_* respectively, where *N_(cc,0)_*  =  aaCgaagagCTaagCCgCCgaaTC and *A_(cc,0)_*  =  gaCTgagaggTTggag. The oligonucleotide GCAT in *V_(cc,0)_* represent the unit *%*, as shown in Table 2.

**Table 2:** The oligonucleotides representing the vertex properties.

Vertex	Oligonucleotides	Denotation
start	5′-ggTaagagaTTCgaCCaCTCaCgagCCaaggTgTCTaaCagTCTgCag-3′	*V_start_*
(cty,GZ)	5′-aCCggaTTgTCCgCaggCCTTggCTCGATGCAaTagaCCTaCgTTaCa-3′	*V_(cty,GZ)_*
(qa,E)	5′-gaTaagaaaTTCaagTgTTggagTTCGATGCAaaCggagagTgagTaT-3′	*V_(qa,E)_*
(qa,null)	5′-gaTaagaaaTTCaagTgTTggagTTGCATGCAaaCggagagaCagaag-3′	*V_(qa,null)_*
(cc,0)	5′-aaCgaagagCTaagCCgCCgaaTCCATGGCATgaCTgagaggTTggag-3′	*V_(cc,0)_*
(cc,null)	5′-aaCgaagagCTaagCCgCCgaaTCTGCATGCAgaCagagaggTaggag-3′	*V_(cc,null)_*
end	5′-ggTaaggaggTaggagagTaaggagCCggTgCgCCaCCTggTTggTaa-3′	*V_end_*

**Figure 5:**
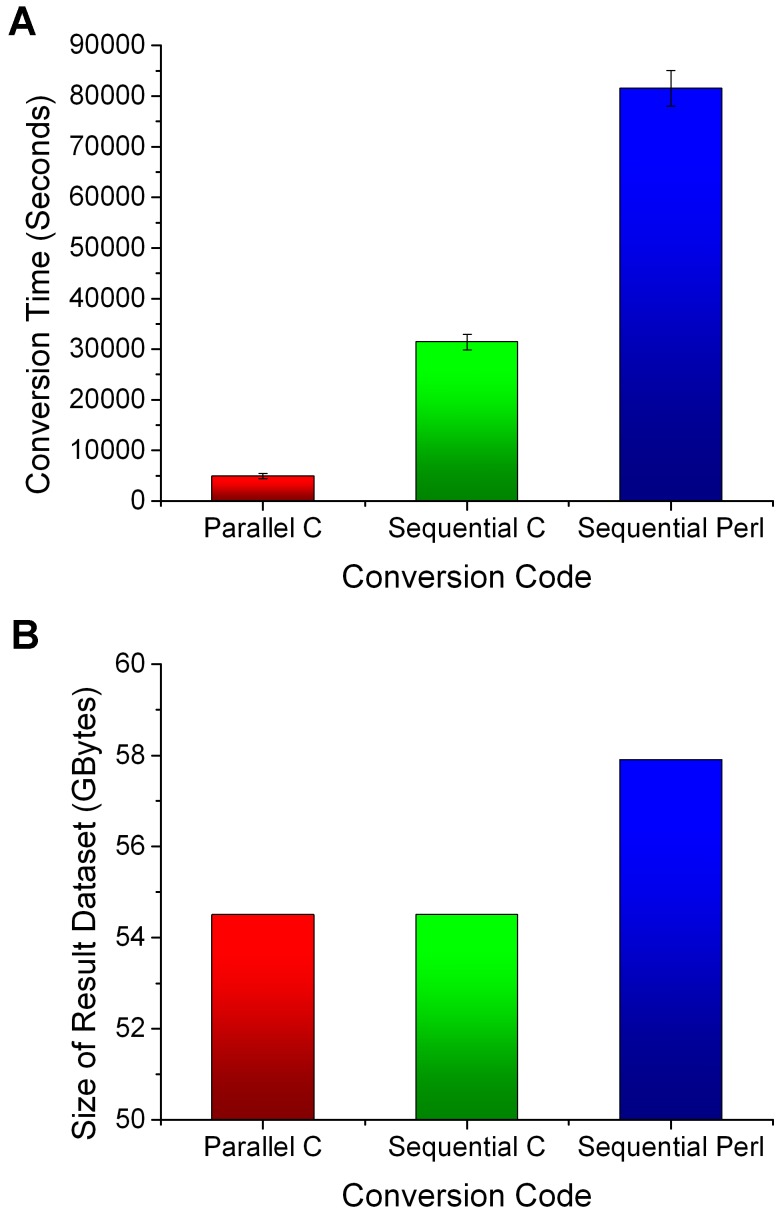
Conversion performance on the test dataset. The result dataset contain DNA sequence information corresponding to the test data. (A) The conversion time is about 4,937 seconds, 31,426 seconds and 81,536 seconds for three programming languages. Error bars depict Standard Error of the mean. (B) The sizes of the datasets are both 54.51 GB for the sequential C and the parallel C. The size is 57.89 GB for the Perl program because the code uses different data block size.

Since the volume of electronic data expands rapidly, it is important to choose the optimal computer architecture for converting big data set. Conversion solutions range from cluster-based computing [37] to cloud-based computing [38]. Considering the cost-effective way to achieve a supercomputer performance, we use the cluster computing. All the conversion experiments in this paper were carried out in the HPC-JNU cluster system. The description about the HPC-JNU is provided in the Materials and Methods section. The sequential and parallel codes in C language are provided in File S3 and File S4 respectively. To evaluate the performance of these conversion programs, our semantic data are partly from the Rest dataset in BTC2012 dataset (http://km.aifb.kit.edu/projects/btc-2012/rest/). This dataset is encoded in NQuads format [39] and includes three data files that range in size from 409.99 MB to 2.69 GB. Figure 5 shows the conversion results of 4.34 GB source dataset in the HPC-JNU cluster system. As an explanatory scripting language, the Perl language has poor IO disk performance. The result of the parallel method shows the best performance although the user of the cluster system has a maximum limit of 80 cores.

### DNA's storage density

At present, remote sensing data are dramatically increasing in volume. For example, the U.S. National Climatic Data Center holds the world's largest archive of weather data and has archived 3 PB (petabyte) satellite imagery [40]. The extreme compactness of DNA is incredible. Because the mean molecular weight of a nucleotide is 330 g/mol [41] and a 200 bp encodes 128 bits in our encoding method, one gram of DNA can store 5.84×10^20^ bits. We approximate DNA's density to water's density (10^−3^ g/mm^3^), then the volume of all DNA sequences encoding 3 PB data is 4.63×10^−2^ mm^3^. We compare favorably contemporaneous storage technologies in Table 3 [42]–[50]. DNA storage has obviously the potential of storing data 100 times more compactly than other technologies.

**Table 3:** Storage volume calculations for 3

**Medium type**	**Year**	**Volume (mm^3^)**	**Notes**
CD-ROM [42]	1982	6.24×10^10^	1.2 mm thickness, 120 mm diameter, 700 MB
DVD-R (single layer) [43]	1996	9.08×10^9^	1.2 mm thickness, 120 mm diameter, 4.7 GB
Blu-ray (single layer) [44]	2002	1.71×10^9^	1.2 mm thickness, 120 mm diameter, 25 GB
Flash memory [45]	2013	1.25×10^8^	72 mm×26.94 mm×21 mm, 1 TB
Magnetic tape (LTO-6) [46]	2012	8.02×10^7^	6.1 µm thickness, 846 m length, 12.65 mm width, 2.5 TB
Hard disk [47]	2013	1.98×10^5^	10 TB/inch^2^, platter 1 mm thickness
Quantum storage [48]–[50]	2012	5.16	5×7 bit/10×10 nm^2^ on the Cu(111) surface, the average height of Cu(111) terrace 65 nm, bilayer cobalt nano-islands 0.8 nm, two additional capping layer 1 nm
This paper	2013	4.63×10^−2^	

### Semantic fusion based on DNA

**Figure 6:**
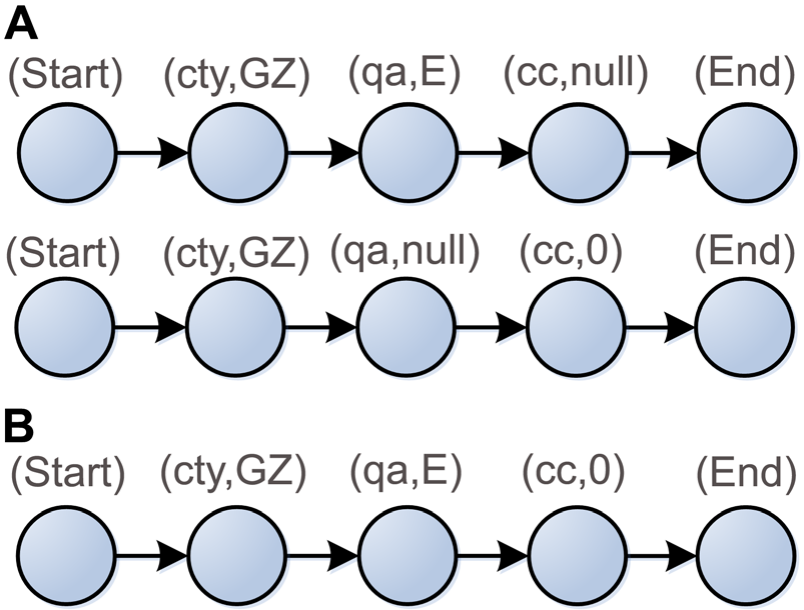
Semantic fusion pattern of an RSI. (A) Two owners of the RSI *E1EB7* select different properties to annotate it. One of them selects the properties *cty* and *qa*. The other selects the properties *cty* and *cc*. The property value *null* means the unannotated property. Certainly, both its data type and its unit are *undefined*. (B) The result property string after semantic fusion represents the complete semantic information of this RSI.

Semantic fusion is the key operation that ontology technology supports. It can automatically implement the union of the properties and semantic relationships. A resource, such as an RSI, and its replicas may be widely distributed over several image replicas databases. The owners of the resource may select different kinds of feature properties to annotate this RSI. We must merge these properties and relationships in order to improve the efficiency and accuracy of the knowledge. As shown in Figure 6, the semantic fusion enables image's semantic information from disparate data sources to be merged. The initial properties dissolve in the new properties and do not preserve their duplicate internal structures. However, the performances of ontology fusion and reasoning degrade rapidly as data grows. Therefore, we build a semantic fusion model based on DNA.

**Figure 7:**
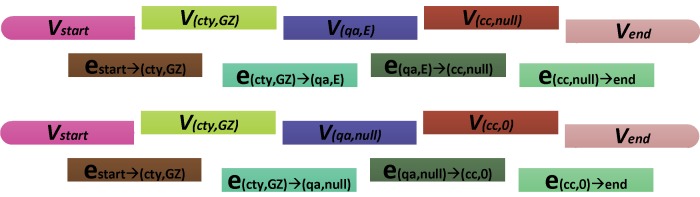
The oligonucleotides in the hybridization and ligation reaction. For each property *i* including the labels *start* and *end*, a 48-nt oligonucleotide *V_i_* is generated. For each edge ij, an oligonucleotide e_ij_ is derived from the 3′ 24-nt of *V_i_* and the 5′ 24-nt of *V_j_*.

Table 2 shows a set of oligonucleotides representing the possible properties labeling the vertices in Figure 6A. As regards orientation, all of the oligonucleotides are written 5′ to 3′. Now each *V* in Figure 6A is divided into two oligonucleotides, each of length 24: *V*  =  *V’V’’*. *V’* and *V’’* are the first and second half of *V*. An edge from the vertex i to the vertex j is encoded as a 48-nt oligonucleotide, obtainable as the Watson-Crick complement of the second and the first halves of the oligonucleotides encoding the vertices i and j touching the edge. For example, the encoding of an edge from the vertex (cty,GZ) to the vertex (qa,E) is given: e_(cty,GZ)→(qa,E)_  =  AGCTACGTTaTCTggaTgCaaTgTCTaTTCTTTaagTTCaCaaCCTCa. For every vertex and every edge in Figure 6A, large quantities of *V_i_* and e_ij_ are mixed together in the hybridization and ligation reaction as shown in Figure 7. The oligonucleotides *V_i_* served as splints to bring oligonucleotides associated with compatible edges together for ligation. Consequently, many DNA molecules encoding the property string are created. The remaining steps, as well as the conclusion in the output, are filtering and screening procedures. We use the Adleman style [1], [51] algorithm for obtaining the result property string:

Input: DNA molecules generated randomly in large quantities.

- Step 1: Reject all DNA molecules that do not begin with *V_start_* and end in *V_end_*.
- Step 2: Reject all DNA molecules encoding property strings that do not involve exactly 5 vertices.
- Step 3: Reject all DNA molecules that contain the oligonucleotide TGCATGCA encoding the *null* value.

Output: Read out the property strings (if any).

**Figure 8:**
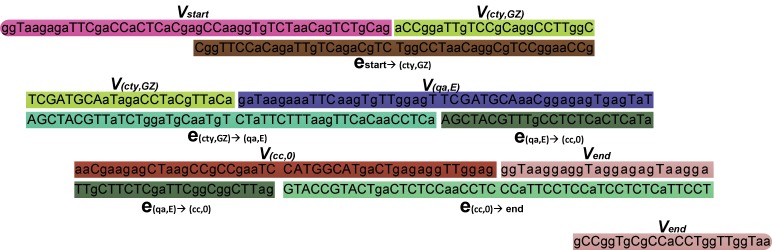
DNA sequence representing the complete semantic information.

As shown in Figure 8, we can obtain the result property string by using the semantic fusion method based DNA. It is consistent with the semantic properties in Figure 6B.

### Abstract representation of semantic fusion

The above algorithm can be formally described by an abstract model. This abstract model is based on the data structure of the tubes. A tube is a multi-set of finite strings over the alphabet {A, C, G, T}, namely the DNA alphabet. Given a tube, one can perform the following operations:

1. pre-separate(*T*, *s*)/post-separate(*T*, *s*)/sub-separate(*T*, *s*). Given a tube *T* and a string *s* over the alphabet {A, C, G, T}, this operation creates a tube containing all strands in *T* that have the string *s* as a prefix/postfix/substring.
2. length-separate(*T*, *n*). Given a tube *T* and integer *n*, this operation creates a tube containing all strands in *T* with length less than or equal to *n*.
3. detect(*T*). Given a tube *T*, this operation outputs true if *T* contains at least one DNA molecule, otherwise outputs false.

In our model, each of the oligonucleotides in *T* is of length 48. Thus,

SemanticFusion(*T*):

1. input(*T*)
2. *T* ← pre-separate(*T*, *V_start_*)
3. *T* ← post-separate(*T*, *V_end_*)
4. *T* ← length-separate(*T*, 240)
5. *T* ← sub-separate(*T*, TGCATGCA)
6. detect(*T*).

This model starts with the input tube *T*, containing the result of the ligation reaction. All separate operations select the oligonucleotides and thus require the amplification of the resulting tubes by the PCR (polymerase chain reaction).

Indeed, semantic fusion problem have been shown to be an NP-complete problem [52], [53], which means that it is unlikely to find an algorithm working in polynomial time. The semantic fusion on image properties of modest size requires an altogether impractical amount of time on conventional electronic computer [54], [55]. However, we use a finite sequence of ligation reaction and screening operations described above to solve the semantic fusion problem. A fusion starts with an initial tube and ends with one final tube. The fusion time depends solely on the total time of ligation reaction and five screening steps instead of the number of semantic properties and ontology complexity. Then the massive parallelism of DNA renders exponential time complexity in semantic fusion to linear time.

## Conclusions

Semantic fusion is a process that is ubiquitous in nature. In this paper, a novel DNA-based semantic fusion model is proposed. The model combines organically parallel strategy with DNA encoding, which makes semantic conversion more efficient and storage density higher. Furthermore, we describe the abstract representation of semantic fusion and thus show that the fusion time of semantic properties in remote sensing images depends solely on the biochemical reactions and operations instead of the ontology. However, there are still many issues to be considered. Foremost issue is error. DNA molecules are fragile and they break easily. The errors of separate operations with DNA strands can make a really dramatic difference. Thus, steps towards coping with errors should be taken in. In future work, we also implement the ligation reaction and screening procedures based on biochemical techniques and clarify details in another paper.

## Materials and Methods

### Mapping from semantic information to an oligonucleotide

All properties and property values are converted to binary strings based on ASCII encoding. Each character corresponds to an 8-bit binary code. For example, the property *cty* has the binary code *011000110111010001111001*. Conversion code in File S4 can then convert these bits to a or g for 0 and T or C for 1. Bases are chosen randomly according to the result of function *rand()*. Considering the big dataset, we add a 32-bit address starting from *00000000000000000000000000000000*. For example, the properties and property values of an RSI *E1EB7* in Figure 3 is represented by the string *startctyGZ qa E cc00 end*, where the symbol represents a whitespace character, *start* and *end* are the labels of the new vertices added in Figure 4. This property string has an ASCII code *001000000111001101110100011000010111001001110100011000110111010001111001010001110101101000100000011100010110000100100000010001010010000001100011011000110011000000110000001000000010000000100000011001010110111001100100*. It is then encoded to two 200 nt oligonucleotides by the conversion code given in File S4. Each encodes a 128-bit data block (128 nt). Before synthesized, the sequence is augmented to include the bases representing data type and data unit. For example, an oligonucleotide *aCCggaTTgTCCgCaggCCTTggCaTagaCCTaCgTTaCa* is the result of encoding the property *ctyGZ* in the vertex (cty,GZ). Considering the data type is *string* and data unit is *undefined*, we add TCGA and TGCA to the original oligonucleotide according to Table 1. Thus, the final oligonucleotide of the vertex (cty,GZ) is *aCCggaTTgTCCgCaggCCTTggCTCGATGCAaTagaCCTaCgTTaCa*, as shown in Table 2.

### Specification of the cluster system

The HPC-JNU cluster system (http://hpc.jnu.edu.cn/) has 20 computational nodes. Each node is connected via the InfiniBand network. Table 4 shows the specifications of the HPC-JNU cluster system. Figure S1 and Figure S2 show the photographs of the computational nodes and the storage node.

**Table 4:** Specifications of the HPC-JNU cluster system.

Hardware			Software	
	Computational node	Storage node		
CPU	AMD Opteron 2.4 GHz	Intel Xeon 2.13 GHz	OS	CentOS 6.2
Number of nodes	20	1	MPI	Open MPI 1.6
Number of CPU cores/node	24	4	File System	NFS 4.1
Number of CPU cores	480	4	Queue Scheduler	Torque 3.3
Memory/node	48 GB	8 GB		
Disk	300 GB	26TB RAID5 Array		
Interconnection network	40 G QDR InfiniBand	40 G QDR InfiniBand		

## Supporting Information

Figure S1 **Photograph of the computational nodes.** (JPG).
(JPG)

Figure S2 **Photograph of the storage node.** (JPG).
(JPG)

File S1 **Code for remote sensing data ontology (see also** http://cs.jnu.edu.cn/sun/ontology **).** Computer code in the RDF Schema language is used to generate the remote sensing data ontology in **Figure 1**. The RDF/OWL API is required. (RDFS).
(RDFS)

File S2 **Code for ID 103001001E1EB700 instance (see also** http://cs.jnu.edu.cn/sun/ontology **).** Computer code in the RDF language is ontology annotation file of remote sensing data (catalog ID 103001001E1EB700) instance in **Figure 2**. The RDF/OWL API is required. (RDF).
(RDF)

File S3 **The sequential conversion code in C language.** The code accesses and converts the data stored contiguously on disk. Despite the cache provided by the operating system, an application that performs a large number of reads, conversions and writes usually faces the performance challenge. GCC compiler is required. (C).
(C) Click here for additional data file.

File S4 **The parallel conversion code in C language.** To support the run-time allocation of conversion tasks, a manager/worker-style parallel C program has been built. The multiple processes of this parallel program can simultaneously access and convert big data by utilizing the MPI-IO. The MPI API is required. (C).
(C) Click here for additional data file.

## References

[1] Adleman LM (1994) Molecular computation of solutions to combinatorial problems. Science 266: 1021–1024. 7973651 10.1126/science.7973651

[2] Lipton R (1995) DNA solution of hard computational problems. Science 268: 542–545. 7725098 10.1126/science.7725098

[3] Bancroft C, Bowler T, Bloom B, Clelland CT (2001) Long-term storage of information in DNA. Science 293: 1763–1765. 10.1126/science.293.5536.1763c 11556362

[4] Renear A, Palmer C (2009) Strategic reading, ontologies, and the future of scientific publishing. Science 325: 828–832. 19679805 10.1126/science.1157784

[5] Yoder MJ, Miko I, Seltmann KC, Bertone MA, Deans AR (2010) A gross anatomy ontology for hymenoptera. PLoS ONE 5(12): e15991. 21209921 10.1371/journal.pone.0015991 PMC3012123

[6] Janowicz K (2012) Observation-driven geo-ontology engineering. Trans GIS 16: 351–374.

[7] Alterovitz G, Xiang M, Hill D, Lomax J, Liu J, et al (2010) Ontology engineering. Nat Biotechnol 28: 128–130. 20139945 10.1038/nbt0210-128 PMC4829499

[8] Iribarne L, Padilla N, Asensio JA, Criado J, Ayala R, et al (2011) Open-environmental ontology modeling. IEEE Trans Syst Man Cybern A Syst Hum 41: 730–745.

[9] Hastings J, Chepelev L, Willighagen E, Adams N, Steinbeck C, et al (2011) The chemical information ontology: Provenance and disambiguation for chemical data on the biological semantic web. PLoS ONE 6(10): e25513. 21991315 10.1371/journal.pone.0025513 PMC3184996

[10] Ashburner M, Ball C, Blake J, Botstein D, Butler H, et al (2000) Gene ontology: Tool for the unification of biology. Nat Genet 25: 25–29. 10802651 10.1038/75556 PMC3037419

[11] Hey T, Trefethen A (2005) Cyberinfrastructure for e-Science. Science 308: 817–821. 15879209 10.1126/science.1110410

[12] Ma XG, Carranza EJ, Wu CL, Meer FD (2012) Ontology-aided annotation, visualization, and generalization of geographic time-scale information from online geographic map services. Comput Geosci 40: 107–119.

[13] Gerstein M (2012) Genomics: ENCODE leads the way on big data. Nature 489: 208. 10.1038/489208b 22972285

[14] Mervis J (2012) Agencies rally to tackle big data. Science 336: 22. 22491835 10.1126/science.336.6077.22

[15] Lynch C (2008) Big data: How do your data grow. Nature 455: 28–29. 18769419 10.1038/455028a

[16] Jones M, Schildhauer M, Reichman O, Bowers S (2006) The new bioinformatics: Integrating ecological data from the gene to the biosphere. Annu Rev Ecol Evol Syst 37: 519–544.

[17] Woodcock CE, Allen R, Anderson M, Belward A, Bindschadler R, et al (2008) Free access to Landsat imagery. Science 320: 1011. 10.1126/science.320.5879.1011a 18497274

[18] Williams G, Weaver J, Atre M, Hendler J (2010) Scalable reduction of large datasets to interesting subsets. J Web Semant 8: 365–373.

[19] Schneider T, Hashemi A, Bennett M, Brady M, Casanave C, et al (2012) Ontology for big systems: The ontology summit 2012 communique. Appl Ontol 7: 357–371.

[20] Church GM, Gao Y, Kosuri S (2012) Next-generation digital information storage in DNA. Science 337: 1628. 22903519 10.1126/science.1226355

[21] Ke YG, Ong LL, Shih WM, Yin P (2012) Three-dimensional structures self-assembled from DNA bricks. Science 338: 1177–1183. 23197527 10.1126/science.1227268 PMC3843647

[22] Halvorsen K, Wong WP (2012) Binary DNA nanostructures for data encryption. PLoS ONE 7(9): e44212. 22984477 10.1371/journal.pone.0044212 PMC3439488

[23] Borresen J, Lynch S (2012) Oscillatory threshold logic. PLoS ONE 7(11): e48498. 23173034 10.1371/journal.pone.0048498 PMC3500268

[24] Bryant B (2012) Chromatin computation. PLoS ONE 7(5): e35703. 22567109 10.1371/journal.pone.0035703 PMC3342293

[25] Tsuboi Y, Ibrahim Z, Ono O (2005) DNA-based semantic memory with linear strands. Int J Innov Comput I 1: 755–766.

[26] Xu J, Qiang XL, Yang Y, Wang BJ, Yang DL, et al (2011) An unenumerative DNA computing model for vertex coloring problem. IEEE T Nanobiosci 10: 94–98. 10.1109/TNB.2011.2160996 21742570

[27] Sun H, Li SX, Li WJ, Ming Z, Cai SB (2005) Semantic-based retrieval of remote sensing images in a grid environment. IEEE Geosci Remote Sens Lett 2(4): 440–444.

[28] Konrath M, Gottron T, Staab S, Scherp A (2012) SchemEX: Efficient construction of a data catalogue by stream-based indexing of linked data. J Web Semant 16: 52–58.

[29] Hendler J (2003) Science and the semantic web. Science 299: 520–521. 12543958 10.1126/science.1078874

[30] Wang XS, Gorlitsky R, Almeida JS (2005) From XML to RDF: How semantic web technologies will change the design of omic standards. Nat Biotechnol 23: 1099–1103. 16151403 10.1038/nbt1139

[31] Tsou M (2004) Integrating web-based GIS and image processing tools for environmental monitoring and natural resource management. J Geogr Syst 6: 155–174.

[32] Wei YX, Di LP, Zhao BH, Liao GX, Chen AJ (2007) Transformation of HDF-EOS metadata from the ECS model to ISO 19115-based XML. Comput Geosci 33: 238–247.

[33] Batchellera J, Reitsma F (2010) Implementing feature level semantics for spatial data discovery: Supporting the reuse of legacy data using open source components. Comput Environ Urban Syst 34: 333–344.

[34] Geneves, Pierre G, Nabil L, Vincent Q (2011) Impact of XML schema evolution. ACM Trans Internet Technol 11: 1–27.

[35] Gibson DG, Glass JI, Lartigue C, Noskov VN, Chuang RY, et al (2010) Creation of a bacterial cell controlled by a chemically synthesized genome. Science 329: 52–56. 20488990 10.1126/science.1190719

[36] Clelland CT, Risca V, Bancroft C (1999) Hiding messages in DNA microdots. Nature 399: 533. 10376592 10.1038/21092

[37] Afek Y, Alon N, Barad O, Hornstein E, Barkai N, et al (2011) A biological solution to a fundamental distributed computing problem. Science 331: 183–185. 21233379 10.1126/science.1193210

[38] Fox A (2011) Cloud computing-what’s in it for me as a scientist. Science 331: 406–407. 21273473 10.1126/science.1198981

[39] Cyganiak R, Harth A, Hogan A (2012) N-quads: Extending n-triples with context. Available: http://sw.deri.org/2008/07/n-quads/. Accessed 2012 Nov 29.

[40] Lattanzio A, Schulz J, Matthews J, Okuyama A, Theodore B, et al (2013) Land surface albedo from geostationary satellites. B Am Meteorol Soc 94: 205–214.

[41] Kneuer C, Sameti M, Bakowsky U, Schiestel T, Shirra H, et al (2000) A nonviral DNA delivery system based on surface modified silica-nanoparticles can efficiently transfect cells in vitro. Bioconjug Chem 11: 926–932. 11087343 10.1021/bc0000637

[42] Imai H (1982) Sony CDP-101 co player. Stereo Review 12: 63.

[43] Mimura H (1997) DVD-video format. Proceedings of IEEE COMPCON 97. San Jose, California, United States: IEEE. 291–294.

[44] Blu-ray Disc Association (2010) White paper blu-ray disc format. Available: http://www.blu-raydisc.com/en/Technical/TechnicalWhitePapers/General.aspx. Accessed 2013 Jul 11.

[45] Kingston (2013) Kingston digital ships its fastest, world's largest-capacity USB 3.0 flash drive. Available: http://www.kingston.com/us/company/press?article=6487. Accessed 2013 Jul 11.

[46] Rivera R, Vargas G, Vazquez M (2012) IBM system storage LTO ultrium 6 tape drive performance white paper. Available: http://public.dhe.ibm.com/common/ssi/ecm/en/tsw03182usen/TSW03182USEN.PDF. Accessed 2013 Jul 11.

[47] Hussain S, Kundu S, Bhatia CS, Yang H, Danner AJ (2013) Heat assisted magnetic recording (HAMR) with nano-aperture VCSELs for 10 Tb/in^2^ magnetic storage density. Proceedings of SPIE 8639, Vertical-Cavity Surface-Emitting Lasers XVII, 863909. San Francisco, California, United States.

[48] Brovko OO, Stepanyuk VS (2012) Quantum spin holography with surface state electrons. Appl Phys Lett 100: 163112.

[49] Oka H, Ignatiev PA, Wedekind S, Rodary G, Niebergall L, et al (2010) Spin-dependent quantum interference within a single magnetic nanostructure. Science 327: 843–846. 20150496 10.1126/science.1183224

[50] Figuera JDL, Prieto JE, Ocal C, Miranda R (1993) Scanning-tunneling-microscopy study of the growth of cobalt on Cu(111). Phys Rev B 47: 13043–13046. 10.1103/physrevb.47.13043 10005522

[51] Paun G, Rozenberg G, Salomaa A (1998) DNA computing: New computing paradigms. Berlin: Springer-Verlag. 43–50 p.

[52] Glimm B, Horrocks I, Lutz C, Sattler U (2008) Conjunctive query answering for the description logic SHIQ. J Artif Intell Res 31: 157–204.

[53] Calvanese D, Giacomo GD, Lembo D, Lenzerini M, Rosati R (2005) DL-lite: Tractable description logics for ontologies. Proceedings of the 20th National Conference on Artificial Intelligence and the 17th Innovative Applications of Artificial Intelligence Conference, AAAI-05/IAAI-05. Pittsburgh, PA, United states. 602–607.

[54] Leida M, Gusmini A, Davies J (2012) Semantics-aware data integration for heterogeneous data sources. J Ambient Intell Humaniz Comput. Available: http://link.springer.com/content/pdf/10.1007%2Fs12652-012-0165-4.pdf. Accessed 2013 Jul 24.

[55] Lewis JJ, Callaghan RJO, Nikolov SG, Bull DR, Canagarajah N (2007) Pixel- and region-based image fusion with complex wavelets. Inf Fusion 8: 119–130.

