# Supplementary figures and images for: A DNA-Based Semantic Fusion Model for Remote Sensing Data
Source: PLoS One. 2013 Oct 8;8(10):e77090. doi: 10.1371/journal.pone.0077090 (PMC3792926; doi:10.1371/journal.pone.0077090)

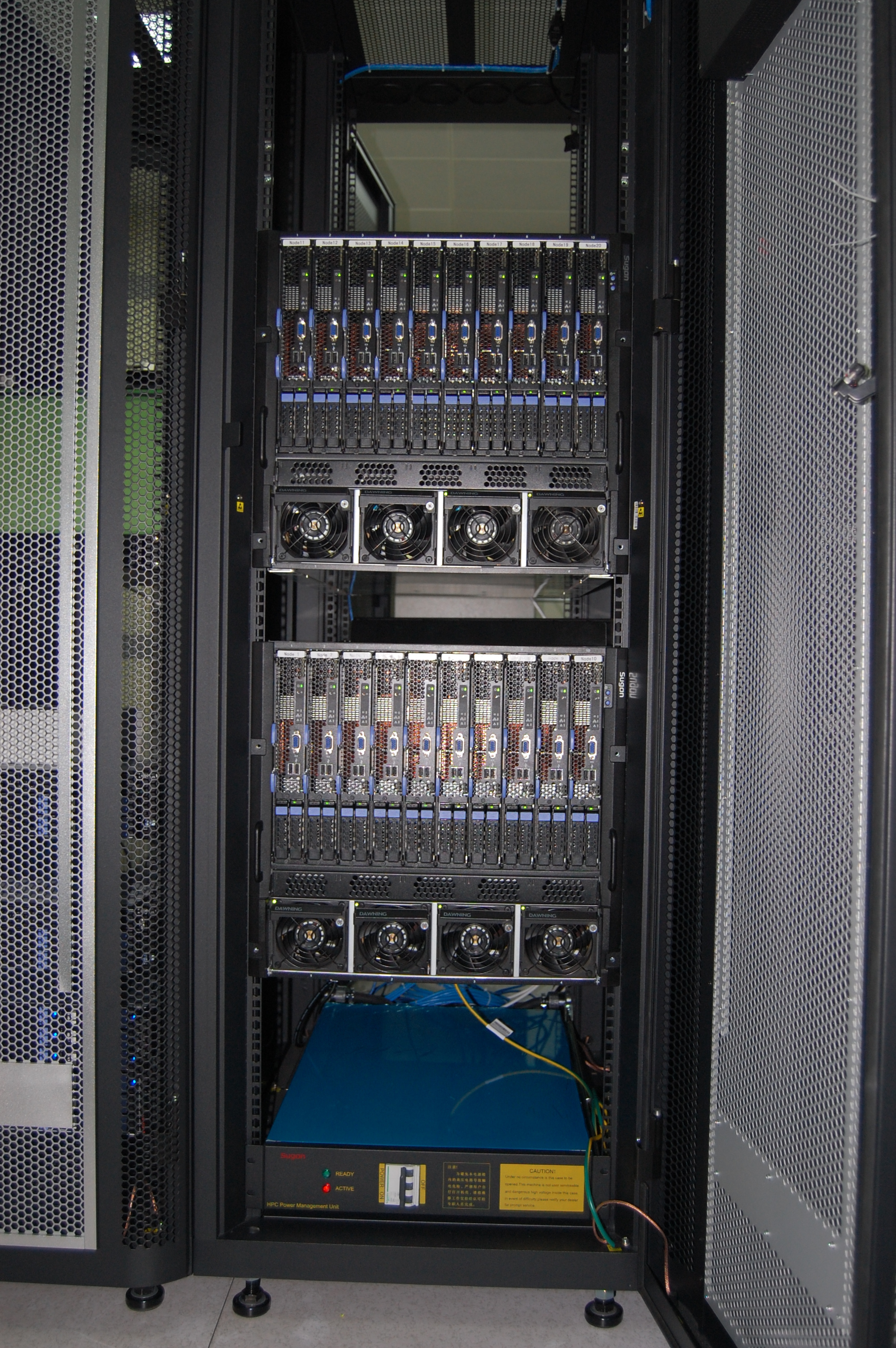

Supplement: Figure S1 — Photograph of the computational nodes. (JPG). (JPG) [file pone.0077090.s001.jpg]

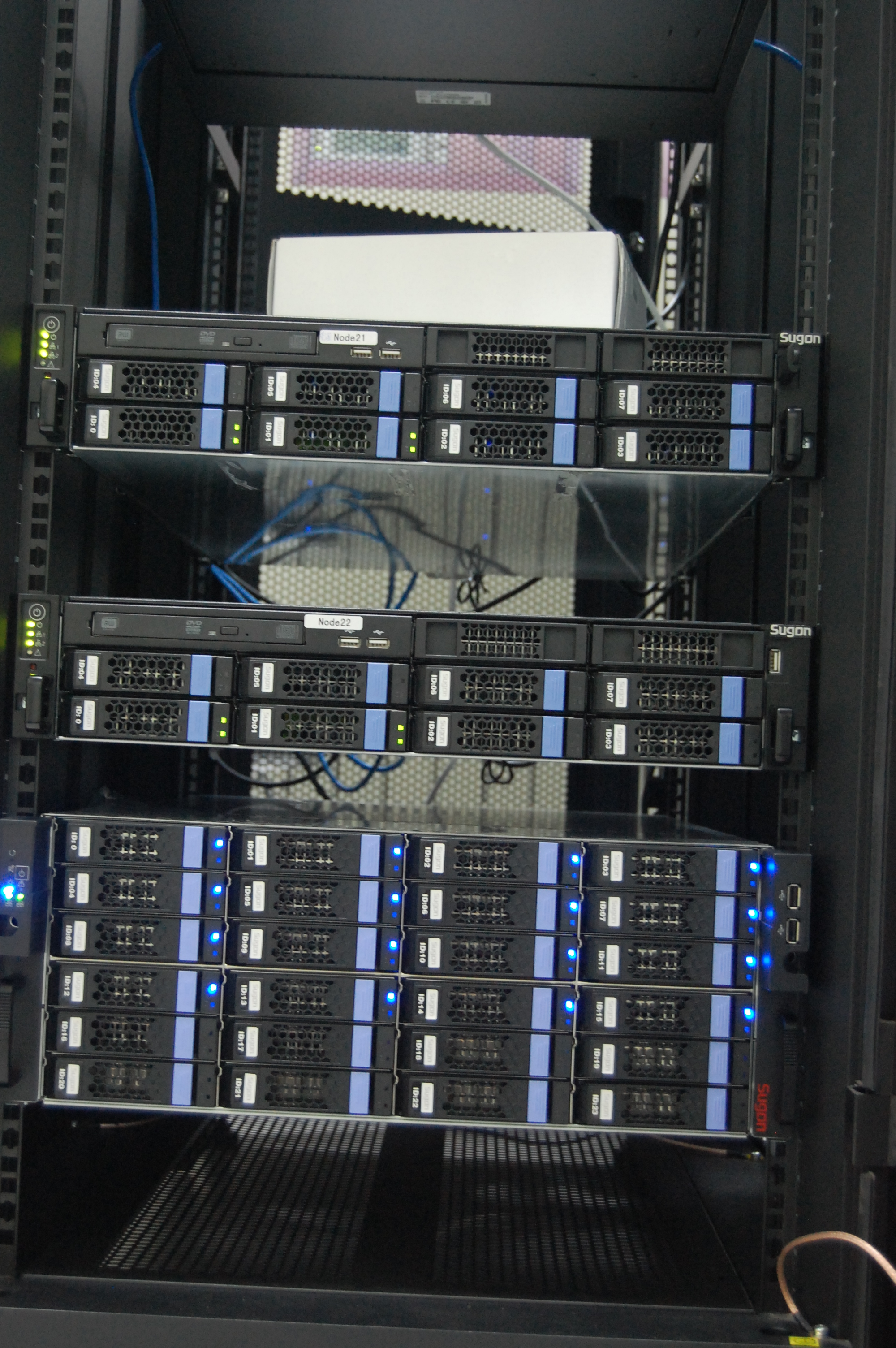

Supplement: Figure S2 — Photograph of the storage node. (JPG). (JPG) [file pone.0077090.s002.jpg]
